# Correlations Between the Bacterial and Fungal Flora and Amino Acid Composition of the Zhuangzu Acid Congee Produced Around the Zuojiang River Basin, Guangxi

**DOI:** 10.3390/foods13233736

**Published:** 2024-11-22

**Authors:** Ao Huang, Qin-Ren Zhang, De-Qiang Xiao, Wei-Sheng Xu, Zu-Lian Bi, Xiu-Die Deng, Xiu-Lian Huang, Jia-Le Song, Quan-Yang Li

**Affiliations:** 1 School of Management, Xiangsihu College of Guangxi Minzu University, Nanning 530225, China; mars.huang@gxxshxy.edu.cn (A.H.);; 2 College of Light Industry and Food Engineering, Guangxi University, Nanning 530004, China; 2116401004@st.gxu.edu.cn; 3 School of Public Health, Guangxi Medical University, Nanning 530021, China; 4 Institute of Nutrition and Health, China Center for Disease Control and Prevention, Beijing 100050, China; 5 Key Laboratory of Environmental Exposomics and Entire Lifecycle Health, Guilin Medical University, Guilin 541199, China

**Keywords:** Zhuangzu acid congee, bacterial diversity, amino acids, rice fermentation, Zuojiang River Basin

## Abstract

In this study, the bacterial, fungal, and amino acid compositions of the Zhuangzu acid congee (ZAC) along the Zuojiang River of Guangxi were analyzed. A total of 14 samples of ZAC were collected in four regions: Fusui (F), Ningming (N), Xiashi (X), and Suxu (S). The predominant bacterial phyla of the four groups were Firmicutes and Proteobacteria. The dominant bacterial genera were *Lactobacillus*, *Bacillus*, *Schleiferitobacillus*, and *Acetobacter*. The fungal alpha diversity in Group S was significantly lower than that in Groups F, X, and N. PCoA clearly distinguished Group S from Groups F, X, and N. The dominant fungal phylum in Group S was Ascomycota (89.12%), whereas those in Groups F, X, and N were Basidiomycota (38.07%), Ascomycota (30.65%), and Fungi (17.39%). The dominant fungal genera in Group S were *Saccharomyces* (42.36%) and *Pichia* (31.66%), whereas those in Groups F, X, and N were *Mortierella* (17.23%) and *Russula* (13.34%). The proportions of the six flavor amino acids were relatively high, and all four groups of samples were above 30.00%. PLS-DA of amino acids demonstrated that all four groups of samples could be significantly distinguished. Additionally, the concentrations of taurine, serine, leucine, phosphoserine, asparagine, 1-methylhistidine, and 3-methylhistidine in Group S were significantly greater than those in Groups F, X, and N. Correlation analysis revealed that 6 bacterial and 15 fungal genera were significantly correlated with amino acids, particularly *Leuconostoc* and *Schleiferlactobacillus,* among bacteria, as well as *Saccharomyces* and *Russula,* among fungi. In conclusion, compared with the disparity in the bacterial community, the disparity in the fungal community was more strongly correlated with the amino acid composition of ZAC. This result indicated that the difference in the fungal community might cause the variation in the amino acid composition of ZAC.

## 1. Introduction

Traditional fermented foods in China have a long history, are diverse, and are frequently eaten [1]. Representative fermented foods include fermented dairy products, fermented kimchi, and fermented cereal, which are flavor-rich and nutrient-rich [2]. In Fusui, Guangxi, there is a traditional grain-based fermented food, Zhuangzu acid congee (ZAC). The origin of ZAC can be traced back to the Northern Song Dynasty [3]. With respect to ZAC, in the process of natural evolution, ancient people learned fermentation to preserve leftover rice. In addition, from the perspective of genetics, the Zuojiang River Basin (a main tributary of the Zhujiang River Basin) was most likely the location where rice was first cultivated [4], which also shows that the Zhuangzu people have a long history of eating rice. Moreover, historically, the people of Inner Mongolia and Shanxi Province have consumed acid congee as part of their traditions [5], but the materials and fermentation processes used differ. The acid congee in Inner Mongolia and Shanxi is a liquid fermented food (fermented for approximately 48 h) [6], whereas ZAC is fermented mainly in the solid state (fermented for approximately 15 d). ZAC was listed as the representative work of intangible cultural heritage in the Guangxi Zhuang Autonomous Region in 2015. ZAC is made mainly of rice and is rich in vitamins, amino acids, and organic acids [2]. Local people in Guangxi like to use ZAC as a dip for chicken or duck meat or as an auxiliary ingredient in cooking dishes [7]. The fermentation of leftover rice is an ancient custom that is highly important for reducing food waste and improving sustainable development [8].

The naturally fermented acid congee is rich in microbial flora and has a relatively high level of microbial diversity in terms of bacterial and fungal composition. The dominant bacteria of ZAC are Firmicutes and Proteobacteria [9], and the dominant genera are *Lactobacillus* (64.06%) and *Acetobacter* (24.13%) [10]. A comparison of acid congee flora in different regions revealed that the composition of acid congee flora in Shanxi and Inner Mongolia is relatively similar, and its representative bacterium is *Lactobacillus*, whereas in Guangxi, it is *Bacillus* [7]. In terms of fungal flora, *Ascomycota* (97.54%) is the dominant fungal phylum in acid congee, and *Candida* (57.59%) and *Galactomyces* (34.95%) are the dominant fungal genera [5]. Wang et al. [11], and She et al. [12] isolated probiotic lactic acid bacteria and yeasts from acid congee via the pure culture method. Those studies focused on the diversity of bacterial or fungal flora in acid congee, while other studies have explored pure culture methods to identify key strains; however, few reports have undertaken a comprehensive analysis of bacterial and fungal flora in acid congee in detail.

The concentrations of amino acids and organic acids in Guangxi acid congee are significantly higher than those in Shanxi and Inner Mongolia, and the pH value of acid congee in Guangxi is also the highest [7]. The bacterial flora composition of acid congee is significantly related to the content of amino acids and proteins in acid congee [13]. After fermentation, the protein, fat, and ash content of acid congee decreases, whereas the starch and free fatty acid content increases [14]. Acid congee is rich in nutrients, such as macromolecular substances in the rice, which are fermented by bacteria into small-molecule substances and functional components, such as active peptides, polysaccharides, and amino acids, making acid congee easier to digest and absorb [9]. Differences in flora composition also cause changes in sensory indicators, and some lactic acid bacterium strains and acetic acid bacteria have a positive effect on the formation of acid congee flavors [6]. Its special flavor and diverse cuisine pairing are deeply loved by local residents, but few studies have investigated the distribution of ZAC along the Zuojiang River in Guangxi and correlations between its flora and amino acid composition.

ZAC is distributed mainly in southwestern Guangxi along the Zuojiang River Basin; however, there has been little research on the distribution of ZAC along the basin. We assume that the flora and amino acid composition of ZAC distributed along the Zuojiang River Basin are different from those of ZAC found elsewhere. Therefore, this study aimed to investigate the characteristics of the bacterial and fungal community structure of ZAC in the Zuojiang River Basin of Guangxi, determine the amino acid composition of the collected samples, and uncover the correlations between these amino acids and bacterial and fungal communities. This study helps to elucidate the relationship between flora and amino acids in ZAC and provides a theoretical basis for its development and utilization.

## 2. Materials and Methods

### 2.1. ZAC Sample Collection

In the early stage of sampling, we first determined that the areas where ZAC is eaten were mainly distributed along the Zuojiang River Basin. We subsequently designed a sampling plan and selected locations along the Zuojiang River Basin and inland areas not along the Zuojiang River Basin for sampling. To ensure that there was a history of eating ZAC in the local area and the representativeness of ZAC samples, the following inclusion criteria were followed for the sampling sites and collected samples: (1) local farmers can make their own acid congee, and acid congee is sold in the local market; (2) there are Zhuangzu people living there; (3) there is natural solid-state fermented acid congee that can be taken and used as needed; and (4) rice is used as the raw material.

**Figure 1:**
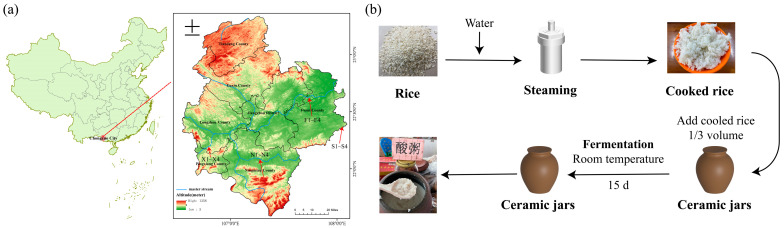
Sampling point distribution and production process of ZAC. (**a**) Distribution map of acid congee consumption along the Zuojiang River Basin. (**b**) Flowchart of traditional acid congee production. Map based on standard map GS (2020) 4619 downloaded from the Standard Map Service website of the Ministry of Natural Resources, without modification.

Fourteen samples of ZAC were collected from farmer households in four counties in the Guangxi Zhuang Autonomous Region from September to November 2023. Four samples were collected from Fusui County (F1–F4), four samples were collected from Ningming County (N1–N4), three samples were collected from Xiashi County (X1–X3), and three samples were collected from Suxu County, an inland area separated from the Zuojiang River (S1–S3). The collected samples were packaged and stored at −80 °C and then used for microbial diversity and amino acid composition assessment. The geographical locations of sampling points are shown by the red arrows in Figure 1a. F, X, and N were distributed along the Zuojiang River, whereas S was located in an inland area distant from the Zuojiang River.

### 2.2. ZAC Sample Production

ZAC is a type of natural fermentation product that has been passed down and fermented in farmers’ homes for more than one thousand years (the result of natural selection) [13]. Although ZAC production of each farmer may vary, the entire production process of ZAC has common characteristics. These characteristics make the production conditions and sanitary conditions of ZAC as consistent as possible. Figure 1b shows the common characteristics of the production process of ZAC. The raw material for making ZAC is rice, which is steamed and left to stand at room temperature. A 1/3 volume of cold cooked rice is subsequently added to the ceramic pot (containing the ready-to-use acid congee), and the ceramic lid is covered for natural solid-state fermentation. Fermentation should be carried out in a natural open environment for approximately 15 days, during which no edible oil should be allowed to contaminate it. After solid-state fermentation, ZAC becomes a thick porridge. When the lid is opened, a strong aroma similar to a rice wine aroma can be smelled.

### 2.3. Bacterial DNA Extraction and 16S rRNA High-Throughput Sequencing in Acid Congee

This step was performed according to the manufacturer’s instructions. Total genomic DNA from acid congee samples was extracted using a TGuide S96 kit (Tiangen Bio, Beijing, China), and the concentration and quality of the DNA were checked with a NanoDrop spectrophotometer (Thermo Scientific, Waltham, MA, USA) and 1.8% agarose gel electrophoresis. The hypervariable region V3–V4 of the 16S rDNA gene was amplified with the primer pair 338F: 5′-ACTCCTACGGGAGGCAGCA-3′ and 806R: 5′-GGACTACHVGGGTWTCTAAT-3′. The PCR amplification program was as follows: 95 °C for 5 min and 30× (95 °C, 30 s; 50 °C, 30 s; and 72 °C, 40 s). The last step was performed at 72 °C for 7 min until the program was stopped. The PCR products were detected by 1.8% agarose gel electrophoresis, and the purified PCR products were collected (Omega, McKinney, TX, USA). Paired-end sequencing (2 × 250 bp) was subsequently performed on the Illumina Novaseq 6000 platform.

### 2.4. Bioinformatic Analysis

The bioinformatic analysis was performed via the QIIME2 platform [15]. After low-quality sequences were removed, USEARCH (version 10.0) was used to construct operational taxonomic units (OTUs) on the basis of 97% similarity. After selecting qualified OTU sequences without chimeras, they were compared with the SILVA database (version 138.1) (confidence threshold of 70%) to determine the classification of OTUs [16]. Alpha diversity and beta diversity were analyzed via QIIME 2 2024.5. One-factor analysis of variance (ANOVA) was used to compare the relative abundance of the bacterial community composition in different acid congee samples. Linear discriminant analysis (LDA) combined with effect size (LEfSe) was used to evaluate the differentially abundant taxa. The online BMK Cloud platform (https://www.biocloud.net) was used to further analyze the correlation content heatmap and beta diversity of all samples at the phylum level.

### 2.5. Determination of Free Amino Acids

Approximately 1.0 g of an acid congee sample was homogenized, 0.5 g of homogenate sample was weighed into a 2 mL Eppendorf tube, and the sample was centrifuged to obtain the supernatant (1000 rpm, 10 min, 4 °C). Subsequently, 0.2 mL of 10% sulfosalicylic acid solution was added to the supernatant, and the reaction was allowed to fully remove the protein. The reaction mixture (1000 rpm, 10 min, 4 °C) was centrifuged to obtain the supernatant, which was filtered through a 0.2 μm organic filter membrane. An S-433D amino acid analyzer (Sykam, Munich, Germany) was used to detect free amino acid levels in the samples. The specific parameters were as follows: chromatographic column: LCA K07/Li; column temperature: 58–74 °C gradient temperature control; injection volume: 50 μL; buffer: lithium citrate, A (pH 2.9), B (pH 4.2), C4 (pH 8.0); flow rate of the buffer system: 0.45 mL/min; flow rate of the derivatization system: 0.25 mL/min; detection wavelength: 570 nm and 440 nm.

### 2.6. Data Analysis

SPSS version 26 was used for data analysis. Significance level was regarded as *p* < 0.05, and the data are expressed as means ± standard deviation. One-way analysis of variance was used to analyze the data that were normally distributed among the four groups, and the Kruskal–Wallis test was used to analyze the data that were not normally distributed among the four groups. Principal component analysis (PCA) was used to evaluate the changes in amino acid composition in each group of samples, in order to visually compare the differences among different groups. Spearman correlation analysis was performed via R Studio to explore the correlations among the top 20 bacterial genera, fungal genera, and differential amino acids. Bar and line charts were drawn with GraphPad Prism 9.5. Adobe Illustrator version 2023 was used to combine pictures and draw flowcharts.

## 3. Results

### 3.1. Diversity of Bacterial Flora in Acid Congee

**Figure 2:**
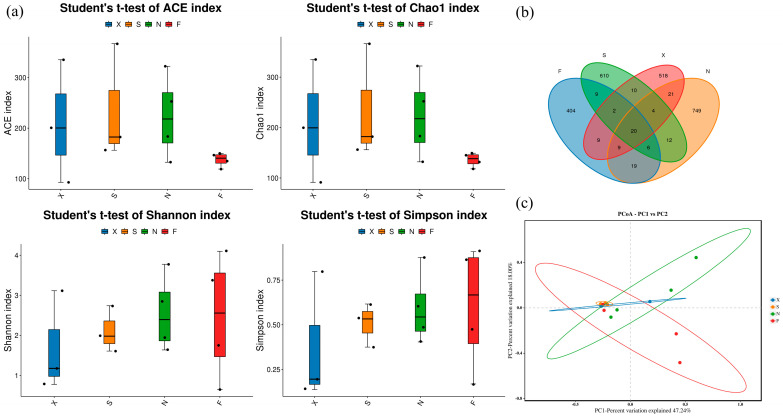
Alpha diversity of acid congee microbiota. (**a**) ACE, Chao1, Shannon, and Simpson indices of four groups of samples. (**b**) Veen graph of four groups of samples. (**c**) PCoA graph of four groups of samples. X: Xiashi, S: Suxu, N: Ningming, F: Fusui.

A total of 1,035,381 pairs of reads were obtained from the sequencing of 14 ZAC samples. After quality control and splicing of the paired-end reads, a total of 943,291 clean reads were generated. Each sample generated at least 36,706 clean reads, and an average of 67,378 clean reads were generated. These sequences were separated into 2402 OTUs on the basis of 97% similarity. On the basis of homologous sequence alignment and information extracted from the SLIVA database, the taxonomic status of the representative sequences in each OTU was determined (Table S1). Figure S1 shows the dilution curves and Shannon curves of all the samples. As the sequencing amount increased, the curves of all acid congee samples tended to flatten, indicating that the sequencing depth basically covered all the species in the samples and could be compared. This method reflects the diversity and distribution of bacteria in a sample well, and the sequencing results represented the true situation of the bacterial community in ZAC. Figure 2a and Table S2 show the α diversity of acid congee samples based on the ACE, Chao1, Shannon, and Simpson indices. It can be seen from the figure that the ACE and Chao1 indices of Group F are lower than those of X, S, and N, but there is no significant difference. The Venn diagram shows the overlap of features between samples by displaying the number of common and unique features. The number of features of the four groups is shown in Figure 2b. The number of features common to the four groups was 20, and the numbers of features unique to the F, S, X, and N groups were 404, 610, 518, and 749, respectively. β diversity was further used to explore the species diversity among samples. Based on Bray–Curtis dissimilarity, PCoA analysis showed that the samples in Groups S and X tended to aggregate and the distance between the two groups was also relatively close, indicating that the species compositions of the two groups of samples were relatively similar. The samples in Groups N and F tended to be scattered, indicating that the species compositions of these two groups of samples were quite different.

### 3.2. Bacterial Flora Structure of Acid Congee

**Figure 3:**
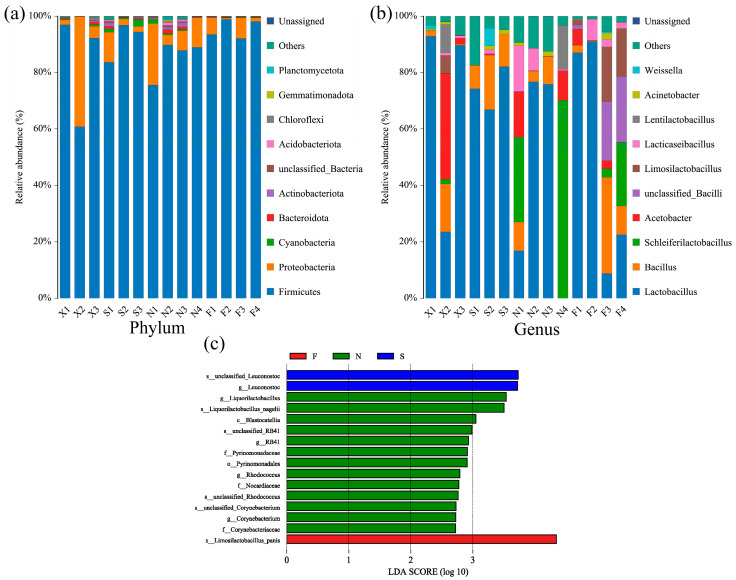
Bacterial flora composition and differential flora of acid congee. Histograms of top 10 relative abundance of bacteria phyla (**a**) and bacteria genera (**b**) in the acid congee samples, with the rest classified as “Others”. (**c**) Histogram of linear discriminant analysis (LDA) scores, where the length of the bars represents the magnitude of the influence of species with significant differences, plotted with a set LDA score ≥ 2.0. X: Xiashi, S: Suxu, N: Ningming, F: Fusui.

Through high-throughput sequencing, a total of 39 phyla were obtained from 14 acid congee samples. Figure 3a shows the composition of the flora in the top 10 levels of acid congee samples, which included Firmicutes, Proteobacteria, Cyanobacteria, Bacteroidota, Actinobacteria, unclassified Bacteria, Acidobacteriota, Chloroflexi, Gemmatimonadota, and Planctomycota. The sequencing database was annotated with species, and the relative abundance of the four groups of acid congee samples at the genus level was greater than 0.1%, resulting in a total of 25 genera. Figure 3b shows the flora composition of the top 10 genera of acid congee samples, which were *Lactobacillus*, *Bacillus*, *Schleiferilactobacillus*, *Acetobacter*, unclassified_*Bacilli*, *Limosilactobacillus*, *Lacticaseibacillus*, *Lentilactobacillus*, *Acinetobacter*, and *Weissella*.

The LEfSe method was used to analyze and screen biomarkers with LDA scores > 2.0. A total of 16 different species were discovered among the four groups. They came from different classes (c), orders (o), families (f), genera (g), and species (s). Specifically, different species included *s__unclassified_Leuconostoc* and *g__Leuconostoc* s_ununclassified_*Leuconostoc* in Group S, *s_L’imosilactobacillus_pnis* in Group F, and *g_L’iquorilactobacillus* in Group N, *s__Liquorilactobacillus_nagelii*, *c__Blastocatellia*, *s__unclassified_RB41*, *g__RB41*, *f__Pyrinomonadaceae*, *o__Pyrinomonadales*, *g__Rhodococcus*, *f__Nocardiaceae*, *s__unclassified_Rhodococcus*, *s__unclassified_Corynebacterium*, *g__Corynebacterium*, and *f_Crynebacteriaceae* (Figure 3c).

### 3.3. Diversity of Fungal Flora in Acid Congee

**Figure 4:**
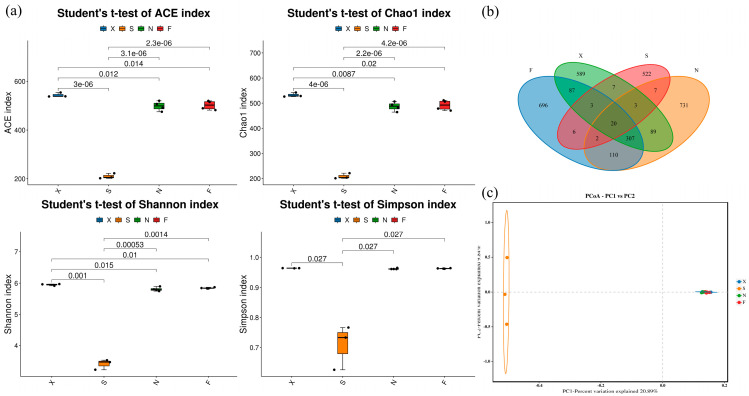
Alpha and beta diversity of fungal flora in acid congee. (**a**) ACE, Chao1, Shannon, and Simpson indices of four groups of samples. (**b**) Veen plots of four groups of samples. (**c**) PCoA plots of four groups of samples.

Figure 4a shows the α diversity of acid congee samples based on the ACE, Chao1, Shannon, and Simpson indices. The figure shows that the ACE, Chao1, Shannon, and Simpson indices of Group S were significantly lower than those of F, X, and N. The ACE, Chao1, and Shannon indices of Group X were significantly higher than those of S, N, and F. The Venn diagram shows the overlap of features between samples by displaying the number of common and unique features. The number of features of the four groups of acid congee samples is shown in Figure 4b. The number of features common to the four groups was 20, and the numbers of features unique to the F, S, X, and N groups were 606, 589, 522, and 731, respectively. PCoA was further used to explore the species diversity among the samples. Figure 4c shows the Bray–Curtis β diversity of the four groups of acid congee samples. The figure shows that the samples in Groups F, X, and N all tended to aggregate and could not be distinguished. The samples in Group S were significantly separated from those in F, X, and N, indicating that the species compositions of the samples in Group S were quite different.

### 3.4. Fungal Flora Structure of ZAC

**Figure 5:**
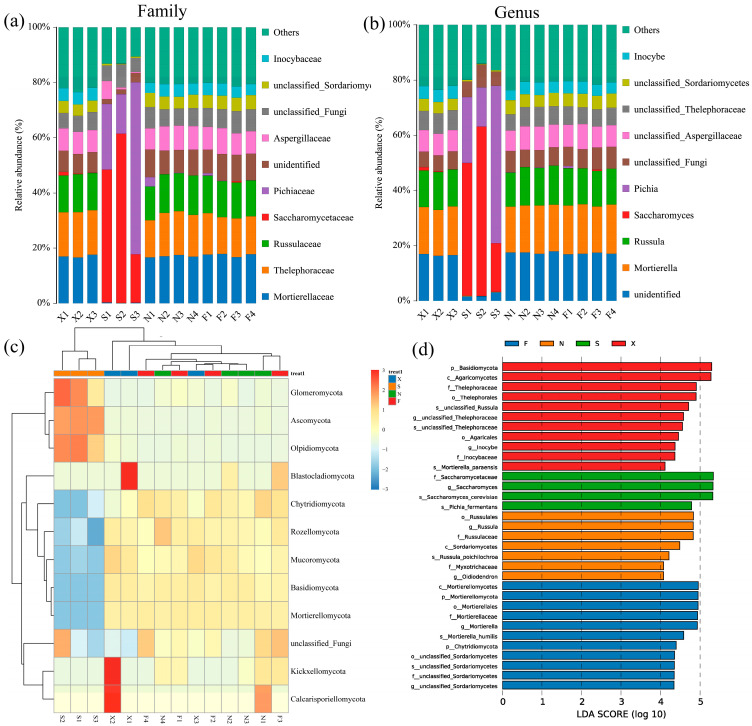
Composition and differential bacterial flora of acid congee fungi. Relative abundance histogram of the top 10 bacterial families (**a**) and bacterial genera (**b**) in acid congee samples. The rest are designated “Others”. (**c**) Relative abundance heat map of bacterial phyla. (**d**) Histogram of linear discriminant analysis (LDA) scores. The length of the bars indicates the influence of species with significant differences. The LDA scores were set to ≥4.0.

A total of 12 phyla were obtained from 14 acid congee samples through high-throughput sequencing. The content heatmap of each sample at the phylum level is shown in Figure 5c. Among them, Ascomycota was the dominant bacterial phylum in Group S, and its relative abundance was significantly higher than that of F, X, and N. In contrast, the relative abundance of Basidiomycota and Mortierellomycota in Groups F, X, and N was significantly higher than that in S. Figure 5a shows the composition of the bacterial community in the top 10 families of the acid congee sample, which were Mortierellaceae, Thelephoraceae, Russulaceae, Saccharomycetaceae, Pichiaceae, unidentified, Aspergillaceae, unclassified_Fungi, unclassified_Sordariomycetes, and Inocybaceae. The sequencing database was used for species annotation, and a total of 46 genera were obtained in four groups with a relative abundance greater than 0.1% at the genus level. The top 10 genera identified in the acid congee samples were unidentified, *Mortierella*, *Russula*, *Saccharomyces*, *Pichia*, *unclassified_Fungi*, unclassified_Aspergillaceae, *unclassified_Thelephoraceae*, *unclassified_Sordariomycetes*, and *Inocybe* (Figure 5b). Among them, the dominant genera in the S group were *Saccharomyces* (42.36%) and *Pichia* (31.66%), while the dominant genera in F, X, and N were *Mortierella* (17.23%) and *Russula* (13.34%).

The LEfSe method was used to analyze and screen biomarkers with LDA scores > 4.0. A total of 33 different species were discovered among the four groups. Among them, the LDA scores of p_Basidiomycota, c_Agaricomycetes, and f_Thalephoraceae in Group X and f_Accharomycetaceae, *g_Accharomyces*, and *s_Accharomyces_cerevisiae* in Group S were all greater than 5.0 (Figure 5d).

### 3.5. Detection of Free Amino Acids in Acid Congee

**Figure 6:**
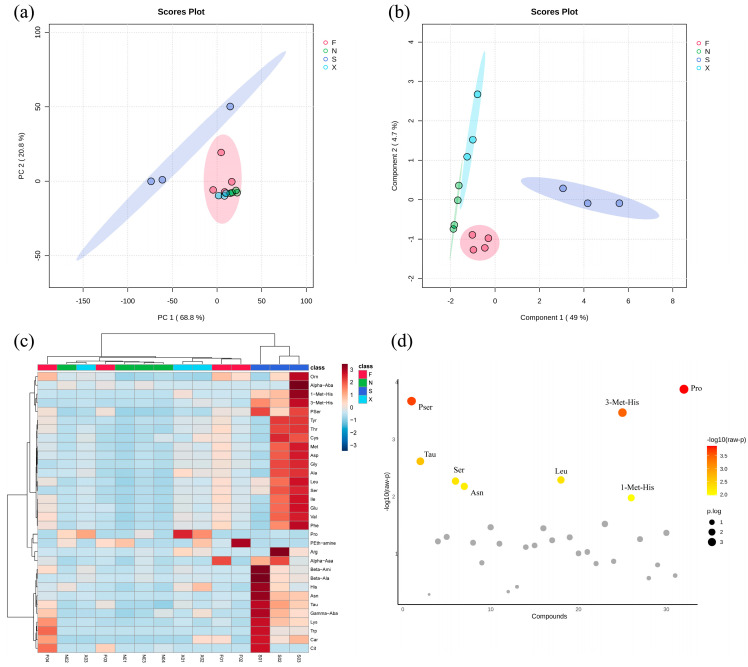
Multivariate statistical analysis of free amino acid composition in acid congee. (**a**) PCA analysis, (**b**) PLS-DA analysis, (**c**) amino acid content cluster heat map analysis, (**d**) one-way ANOVA for significantly different metabolites. FDR *p* value < 0.05 is considered a significant difference. Phosphoserine: Pser, taurine: Tau, serine: Ser, asparagine: Asn, leucine: Leu, 3-methylhistidine: 3-Met-His, 1-methylhistidine: 1-MetHis, and proline: Pro.

A total of 33 free amino acids were detected in all acid congee samples. The average content composition of each group is shown in Table 1. There were six kinds of flavor-producing amino acids, namely, aspartic acid, glutamic acid, glycine, alanine, tyrosine, and phenylalanine, accounting for 35.42% (F), 33.35% (N), 30.77% (S), and 39.59% (X) of the total amino acids. Alanine (11.07 ± 15.31 mg/100 g) and glutamic acid (11.62 ± 14.92 mg/100 g) dominated the flavor-producing amino acids. There were nine essential amino acids, namely, lysine, tryptophan, phenylalanine, methionine, threonine, isoleucine, leucine, valine, and histidine, accounting for 32.04% (F), 24.05% (N), 31.26% (S), and 25.83% (X) of the total amino acids. The highest in content was leucine, followed by lysine and valine. These three essential amino acids are relatively easy for the human body to absorb. Based on multivariate statistical analysis of the amino acid content composition in acid congee, PCA revealed that after dimensionality reduction, two principal components, PC1 (68.8%) and PC2 (20.8%), were obtained, in which F, N, and S were significantly separated (Figure 6a). Furthermore, on the basis of PLS-DA, the four groups clustered together within the group, but were distinguished between the groups, indicating that there were differences among the four groups (Figure 6b). The content clustering heatmap shows the samples in Group S in red, whereas those in Groups F, X, and N are lighter blue (Figure 6c). One-factor analysis of variance showed eight amino acids with significant differences among the four groups, namely, phosphoserine, taurine, serine, asparagine, leucine, 3-methylhistidine, 1-methylhistidine, and proline (Figure 6d). The eight significantly different amino acids among four groups were selected based on the FDR *p* value (Table S3). The differential distribution of the content of these eight amino acids in the four groups is shown in Figure S2. With the exception of proline, the content of the other seven differential abundant metabolites in the S group was significantly higher than that in the F, X, and N groups.

### 3.6. Correlation Analysis Between Bacterial Flora in Acid Congee and Differential Amino Acid Metabolites

**Figure 7:**
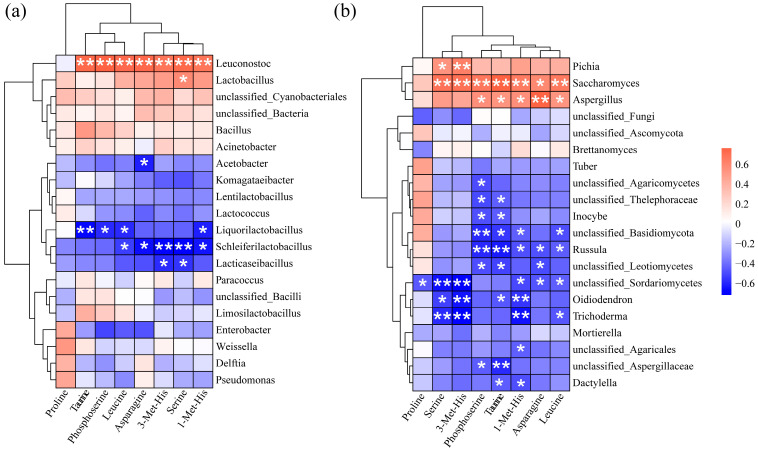
Spearman correlation analysis of acid congee bacterial groups and differential amino acids. (**a**) Spearman correlations between the top 20 bacterial genera and 8 differential amino acids. (**b**) Spearman correlations between the top 20 fungal genera and 8 differential amino acids. Red indicates positive correlation and blue indicates negative correlation. Significance level: * indicates *p* < 0.05, ** indicates *p* < 0.01.

Spearman’s correlation was used to explore the correlations between the top 20 bacterial genera and the 8 differential amino acids (Figure 7a). Five bacterial genera were significantly correlated with differential amino acids. *Leuconostoc* was significantly positively correlated with taurine, phosphoserine, leucine, asparagine, 3-methylhistidine, serine, and 1-methylhistidine. *Lactobacillus* and serine showed a significant positive correlation. *Acetobacter* and asparagine were significantly negatively correlated. *Liquorilactobacillus* was significantly negatively correlated with taurine, phosphoserine, leucine, and 1-methylhistidine. *Schleiferilactobacillus* showed significant negative correlations with leucine, asparagine, 3-methylhistidine, serine, and 1-methylhistidine. *Lacticaseibacillus* showed significant negative correlations with 3-methylhistidine and serine.

Spearman’s correlation was used to explore the correlations between the top 20 fungal genera and the 8 differential amino acids (Figure 7b). There were 15 fungal genera that were significantly correlated with differential amino acids. *Pichia* showed significant positive correlations with serine and 3-methylhistidine. *Saccharomyces* was significantly positively correlated with serine, 3-methylhistidine, phosphoserine, taurine, 1-methylhistidine, asparagine, and leucine. *Aspergillus* was significantly positively correlated with phosphoserine, taurine, 1-methylhistidine, asparagine, and leucine. *Inocybe* was significantly negatively correlated with phosphoserine and taurine. *Russula* was significantly negatively correlated with phosphoserine, taurine, 1-methylhistidine, asparagine, and leucine. *Oidiodendron* was significantly negatively correlated with serine, 3-methylhistidine, 1-methylhistidine, and leucine. *Trichoderma* showed significant negative correlations with serine, 3-methylhistidine, 1-methylhistidine, and leucine. *Dactylella* showed significant negative correlations with taurine and 1-methylhistidine.

## 4. Discussion

In today’s society, fermented foods play an important role in human food choices [1]. Traditional solid-state fermented foods can transform food byproducts into flavorful delicacies, which is highly important for food waste and carbon emissions [8]. Similarly, ZAC, a traditional solid-state fermented food, can transform byproducts (e.g., leftover cooked rice) into acidic porridge with a characteristic flavor. ZAC has a variety of active ingredients that promote health after natural fermentation and is considered a traditional fermented functional food with potential. However, the microbial composition and bioactive components in acid congee have not been fully studied. Therefore, this study collected acid congee samples from four regions in Guangxi along the Zuojiang River to explore the microbial composition and amino acid composition of acid congee samples from different regions. The dominant bacterial genera in the acid congee samples were *Lactobacillus*, *Bacillus*, *Schleiferilactobacillus*, *Acetobacter*, etc. This finding is consistent with a previous study in which high-throughput sequencing was used to study the microbial composition of acid congee in Inner Mongolia, Shanxi, and Guangxi [7]. The results of this study revealed that *Lactobacillus*, *Acetobacter*, *Bacillus*, *Clostridium*, and *Weissella* were the dominant genera in the acid congee from the three regions. The relative abundance of *Lactobacillus* did not differ significantly among the samples from the three regions, but the abundance of *Acetobacter* in the acid congee from Shanxi and Inner Mongolia was significantly higher than that of Guangxi, while *Bacillus* was significantly lower than that from Guangxi. This study also confirmed this finding and revealed that the high relative abundance of *Bacillus* was a characteristic of the bacterial community composition in the acid congee samples from Guangxi.

The metabolic activities in which bacteria participate are important because of the functional active ingredients (amino acids, organic acids, etc.) in acid congee. The probiotic genera *Lactobacillus*, *Bacillus*, and *Schleiferilactobacillus* are important genera involved in metabolic conversion [17]. The dominant strains of *Lactobacillus* and *Bacillus* in Guangxi acid congee samples can ferment to produce lactic acid, whereas the dominant strains of *Acetobacter* in Inner Mongolia and Shanxi samples oxidize ethanol to acetic acid, which in turn is converted into CO_2_ and water [7]. This explains why the concentrations of lactic acid and acetic acid in the Shanxi and Inner Mongolia samples (high abundance of *Acetobacter*) were lower than those in the Guangxi samples (low abundance of *Acetobacter*). Specifically, compared with those in samples from Inner Mongolia acid [6], the relative abundance of *Schleiferillactobacillus* in ZAC was higher. When *Schleiferilactobacillus harbinensis* M1 ferments soy milk, it can produce high concentrations of 2,3-butanedione and acetoin, thereby improving the overall sensory acceptability of fermented soy milk [18]. *Schleiferilactobacillus harbinensis* M1 can convert soybean carbohydrates into lactic acid and acetic acid, convert isoflavone glucosides into aglycones, and hydrolyze soybean protein into oligopeptides and free amino acids. The above studies show that the high abundance of *Schleiferilactobacillus* in ZAC may promote the production of functional active ingredients, providing scientific evidence for the promotion of human health through the consumption of acid congee.

The relative abundance of Ascomycota in Group S in this study was as high as 89.12%, which is similar to the Ascomycota abundance (97.54%) in the Inner Mongolia acid congee [5]. However, the dominant bacterial phyla in Groups F, X, and N were Ascomycota, Basidiomycota, and Mortierellomycota. Moreover, the diversity of the bacterial phylum compositions of F, X, and N further explains why their alpha diversity was significantly higher than that of S. Wang et al. [7] reported that the bacterial community compositions of samples from Shanxi and Inner Mongolia were similar, but significantly different from those of Guangxi samples. The fungal colony composition of Group S was more similar to that of Inner Mongolia, but different from that of the three ZAC samples from the Zuojiang River (F, X, and N). From a geographical perspective, F, X, and N are all located near the Zuojiang River, whereas S, although located in the Zuojiang River Basin, is relatively inland and has regional differences from the first three locations. Therefore, the differences in fungal flora in Group S may be due to geographical isolation.

At the fungal genus level, the dominant genera of Group S were *Saccharomyces* and *Pichia*, whereas the dominant genera of F, X, and N were *Mortierella* and *Russula*. Li Wenya et al. [19] isolated and identified yeasts in acid congee from northern Shanxi during different fermentation periods and reported that *Pichia* and *Saccharomyces* were present throughout the fermentation process. The core fungi associated with acid congee from Inner Mongolia and northwestern Shanxi are dominated by *Pichia* and *Saccharomyces* [5]. The composition of fungi in Group S was more similar to that in the acid congee of Inner Mongolia and different from that in ZAC. The formation of acid congee flavor substances is strongly affected by the bacterial community. Our research revealed that the amino acid composition of Group S was also significantly different from that of F, X, and N. This difference is consistent with the differences in fungal flora. Studies have reported that yeast can affect the bitterness and saltiness of acid congee, but *Pichia* has no positive effect on the flavor quality of acid congee [12]. We further confirmed this through correlation analysis. *Pichia* had a weak positive correlation with differential amino acids, whereas *Saccharomyces* had a strong positive correlation with differential amino acids. Therefore, the correlation between the fungal community and differential amino acids is stronger than that of the bacterial community, among which *Saccharomyces* contributes the most to the formation of amino acids.

The composition and content of amino acids are considered important criteria for evaluating the nutritional value of food [20]. Acid congee contains not only flavor amino acids but also abundant essential amino acids, which play important roles in the presentation of flavor and improvement in the nutritional value of acid congee. Flavor amino acids are mainly divided into three categories: umami, sweetness, and bitterness. Aspartic acid, glutamic acid, serine, and threonine promote the presentation of umami; glycine, alanine, threonine, and valine promote sweetness; and leucine, isoleucine, phenylalanine, valine, lysine, tyrosine, methionine, histidine, tryptophan, and arginine promote bitterness [21]. During the natural fermentation of acid congee, the level of amino acids increases and their nutritional content also increases significantly, resulting in an overall acid taste [14]. Similarly, the proportion of flavor amino acids in the four groups of acidic congee samples was more than 30%, which dominated the overall amino acid ratio. Among them, alanine and glutamic acid were the main compounds, which explains the source of sweetness, umami, and astringency in acid congee [22], indicating that acid congee has unique flavor substances. The essential amino acid content in Guangxi acid congee samples was four to nine times greater than that in Shanxi and Inner Mongolia samples, but the proportion of essential amino acids was lower than that in Shanxi samples [7]. The WHO and FAO recommend that the essential amino acid ratio should be 36%. The essential amino acid ratio in ZAC in this study was between 10.52% and 39.20%, and more than half of the acid congee samples had an essential amino acid ratio of more than 30%, indicating that although the acid congee samples have certain nutritional value, they are not recommended to be consumed alone as a food to supplement amino acids, but rather as a supplement to be consumed with meat such as chicken and duck. Lysine is the limiting amino acid in rice, but the limiting amino acid in acid congee made from rice is not lysine, which indicates that the lysine content increases after fermentation. Lysine is beneficial for maintaining a healthy immune system and normal respiratory health [23]. Therefore, eating ZAC may provide the human body with essential amino acids to enhance immunity and promote human health.

An unsupervised pattern recognition model was established based on PCA [24]. It was observed that Group S could be clearly separated from F, X, and N, and two categories were automatically fitted. The F, X, and N samples could basically be grouped into one group, in which X and N overlapped with each other, reflecting the similarity of the amino acid composition of acid congee samples in some respects. Group S was independently grouped into one group, which was consistent with the results of the fungal flora, clearly reflecting that the amino acid composition of Group S was different from those of the other three groups of acid congee samples. Supervised pattern recognition analysis based on PLS-DA revealed the aggregation and separation trends among the four groups of samples. Combined with the cross-validation results, these findings indicated that model was reliable and had good fitting accuracy. The concentrations of multiple amino acids in Group S were significantly higher than those in Groups F, X, and N, indicating that differences in bacterial flora may affect the production levels of amino acids in acid congee. Studies have reported that taurine levels decrease during aging and that taurine supplementation can reverse this decline and increase lifespan in animal models (mice, nematodes, and monkeys), indicating that taurine is an important antiaging amino acid [25]. Exogenous supplementation with proline maintains intestinal homeostasis by regulating lymphoid tissue inducer cells and effectively alleviates DSS-induced colitis [26]. Leucine, isoleucine, and valine are known as branched-chain amino acids that help enhance muscle protein synthesis and can serve as biomarkers for dyslipidemia, which is closely related to obesity [27]. Reducing dietary isoleucine and valine significantly improves metabolic function, but reducing leucine has essentially no effect [28]. The combination of low systemic serine levels and a high-fat diet accelerates the development of peripheral neuropathy in mice, and normalizing serine levels through dietary supplementation alleviates neuropathy in diabetic mice [29]. These results indicate that increased functional amino acid content in acid congee has the potential to promote body health and improve host physiology.

Spearman’s correlation analysis clarified the relationships among bacterial flora, fungal flora, and differential amino acids in acid congee. In particular, this study revealed that dominant fungal genera presented stronger correlations with differential amino acids, while the co-occurrence network of bacterial groups presented significant complexity and diversity among them. However, this study also has certain limitations. Few samples were collected, and the potential health-promoting effect of ZAC was not evaluated. Future studies can focus on the exploration of dominant fermentation strains and the development of single-strain or mixed-strain fermented acid congee products to explore the relationships between specific acid congee samples in animal models or human health.

## 5. Conclusions

Natural solid fermented acid congee from different regions of Guangxi has a high diversity of bacterial flora. The bacterial flora structures of 14 ZAC samples were generally similar, but the fungal flora of Group S were significantly different from those of F, X, and N. The dominant bacteria were *Lactobacillus*, *Bacillus*, *Schleiferilactobacillus*, and *Acetobacter*. The dominant fungal genera differed, with *Saccharomyces* and *Pichia* being the main species in S, whereas *Mortierella* and *Russula* were the main species in F, X, and N. On quantitative analysis of amino acids, it was found that the sample of natural fermented acid congee contained a unique flavor and was rich in a variety of flavor amino acids (aspartic acid, glutamic acid, glycine, alanine, and tyrosine) and essential amino acids. Relevant analysis revealed that there was a significant correlation (*p* < 0.05) between bacteria, fungal genera, and amino acids, especially *Leuconostoc* and *Schleiferlactobacillus* in bacteria and *Saccharomyces* and *Russula* in fungi. In addition, compared with the differences in the bacterial community, the difference in fungal genera were more strongly correlated with the amino acid flavor of acid congee, and *Saccharomyces* made the most prominent contribution. This research provides a theoretical basis for understanding the microbial diversity and amino acid composition of acid congee resources and emphasizes the support provided by traditional food in the realm of sustainable food development.

## Figures and Tables

**Table 1:** Amino acid composition and content of acid congee.

Type	Component	Concentrations (mg/100 g)			
		F	N	S	X
Flavor amino acids	Aspartate	3.33 ± 2.07 ^ab^	0.99 ± 0.82 ^b^	13.78 ± 12.06 ^a^	3.33 ± 0.57 ^ab^
	Glutamate	9.70 ± 3.79 ^ab^	1.74 ± 1.84 ^b^	30.21 ± 27.03 ^a^	6.21 ± 1.74 ^b^
	Glycine	1.89 ± 2.22 ^b^	0.45 ± 0.48 ^b^	12.59 ± 10.35 ^a^	2.48 ± 0.45 ^b^
	Alanine	6.59 ± 4.58 ^ab^	2.45 ± 2.63 ^b^	29.83 ± 25.93 ^a^	12.32 ± 3.87 ^ab^
	Tyrosine	2.52 ± 1.43 ^ab^	0.51 ± 0.36 ^b^	9.74 ± 8.44 ^a^	1.77 ± 0.85 ^b^
Essential amino acids	Threonine	2.03 ± 1.28 ^ab^	0.34 ± 0.32 ^b^	6.64 ± 5.51 ^a^	1.69 ± 0.29 ^ab^
	Valine	4.25 ± 2.21 ^ab^	0.99 ± 0.73 ^b^	12.24 ± 10.82 ^a^	3.44 ± 1.03 ^ab^
	Isoleucine	2.73 ± 1.65 ^ab^	0.58 ± 0.50 ^b^	8.44 ± 7.08 ^a^	2.11 ± 0.37 ^ab^
	Leucine	7.39 ± 3.82 ^b^	1.43 ± 1.17 ^b^	25.95 ± 14.58 ^a^	5.25 ± 0.91 ^b^
	Lysine	5.66 ± 8.80 ^ab^	0.53 ± 0.44 ^b^	18.05 ± 12.00 ^a^	1.46 ± 1.11 ^b^
	Tryptophan	0.89 ± 0.32 ^a^	0.00 ± 0.00 ^a^	14.32 ± 19.65 ^a^	0.00 ± 0.01 ^a^
	Methionine	1.40 ± 0.94 ^b^	0.33 ± 0.25 ^b^	5.45 ± 4.25 ^a^	1.30 ± 0.23 ^b^
	Histidine	0.26 ± 0.19 ^a^	0.27 ± 0.42 ^a^	2.17 ± 2.20 ^a^	0.99 ± 0.74 ^a^
	Phenylalanine	2.22 ± 1.34 ^ab^	0.46 ± 0.27 ^b^	6.37 ± 5.98 ^a^	1.72 ± 0.33 ^ab^
Other amino acids	Serine	2.71 ± 2.49 ^b^	0.69 ± 0.67 ^b^	13.98 ± 7.93 ^a^	3.04 ± 0.24 ^b^
	Asparagine	1.22 ± 0.74 ^b^	0.26 ± 0.25 ^b^	18.40 ± 12.74 ^a^	1.01 ± 0.74 ^b^
	α-aminoadipic acid	0.38 ± 0.51 ^a^	0.06 ± 0.08 ^a^	0.69 ± 0.46 ^a^	0.09 ± 0.02 ^a^
	Citrulline	8.75 ± 11.51 ^a^	0.54 ± 1.00 ^a^	11.61 ± 20.11 ^a^	0.00 ± 0.00 ^a^
	Cystine	0.14 ± 0.10 ^ab^	0.06 ± 0.06 ^b^	0.62 ± 0.54 ^a^	0.18 ± 0.09 ^ab^
	Methionine	1.40 ± 0.94 ^b^	0.33 ± 0.25 ^b^	5.45 ± 4.25 ^a^	1.30 ± 0.23 ^b^
	Carnosine	0.08 ± 0.09 ^ab^	0.00 ± 0.00 ^b^	0.20 ± 0.14 ^a^	0.03 ± 0.06 ^b^
	Arginine	0.89 ± 0.32 ^a^	0.61 ± 1.00 ^a^	14.32 ± 19.65 ^a^	4.61 ± 4.11 ^a^
	Phosphoserine	1.13 ± 0.12 ^b^	0.10 ± 0.04 ^b^	3.10 ± 1.26 ^a^	0.34 ± 0.06 ^b^
	Taurine	0.48 ± 0.24 ^b^	0.11 ± 0.06 ^b^	1.54 ± 0.73 ^a^	0.33 ± 0.11 ^b^
	Phosphoethanolamine	0.03 ± 0.05 ^a^	0.01 ± 0.02 ^a^	0.00 ± 0.00 ^a^	0.01 ± 0.02 ^a^
	α-aminobutyric acid	0.00 ± 0.01 ^a^	0.01 ± 0.01 ^a^	0.04 ± 0.07 ^a^	0.00 ± 0.00 ^a^
	β-alanine	0.01 ± 0.01 ^a^	0.04 ± 0.08 ^a^	1.41 ± 1.59 ^a^	0.15 ± 0.07 ^a^
	β-aminoisobutyric acid	0.14 ± 0.19 ^a^	0.03 ± 0.06 ^a^	0.88 ± 1.01 ^a^	0.16 ± 0.08 ^a^
	γ-aminobutyric acid	0.50 ± 0.53 ^b^	0.20 ± 0.25 ^b^	1.96 ± 1.28 ^a^	0.43 ± 0.34 ^b^
	3-methylhistidine	0.01 ± 0.02 ^b^	0.01 ± 0.00 ^b^	0.29 ± 0.13 ^a^	0.04 ± 0.02 ^b^
	1-methylhistidine	0.03 ± 0.02 ^b^	0.00 ± 0.00 ^b^	0.58 ± 0.42 ^a^	0.07 ± 0.09 ^b^
	Ornithine	7.97 ± 3.31 ^a^	1.43 ± 1.48 ^a^	10.81 ± 11.98 ^a^	1.67 ± 2.30 ^a^
	Proline	0.03 ± 0.03 ^b^	2.46 ± 2.96 ^b^	0.62 ± 0.46 ^b^	13.58 ± 4.17 ^a^

Note: Different letters in the same row indicate significant differences, *p* < 0.05.

## Data Availability

The original contributions presented in the study are included in the article and Supplementary Material, further inquiries can be directed to the corresponding authors.

## Supplementary Materials

The following supporting information can be downloaded at https://www.mdpi.com/article/10.3390/foods13233736/s1. Figure S1: Dilution curve (a) and flavor curve (b) of 14 acid congee samples; Figure S2: Amino acids with significant differences among the four groups; Table S1: 16S rRNA sequencing results of 14 acid congee samples and the number of different classification states; Table S2: The α-diversity index in acid congee samples; Table S3: Anova Post-hoc tests.
