# Supplementary material for: Correlations Between the Bacterial and Fungal Flora and Amino Acid Composition of the Zhuangzu Acid Congee Produced Around the Zuojiang River Basin, Guangxi
Source: Foods. 2024 Nov 22;13(23):3736. doi: 10.3390/foods13233736 (PMC11640507; doi:10.3390/foods13233736)
Supplement: Supplementary file 1 [file foods-13-03736-s001.zip › foods-3244675-supplementary.pdf]

Correlation between the bacterial and fungal flora and amino acid composition of the Zhuangzu Acid Congee produced around the Zuojiang River Basin, Guangxi

Ao Huang [1,+], Qinren Zhang [2,+], Deqiang Xiao [3], Weisheng Xu [4], Zulian Bi [1], Xiudie Deng [1], Xiulian Huang [1], Jia-le Song [5,*] and Quanyang Li [2,*]

1. School of Management, Xiangsihu College of Guangxi minzu University; Nanning 530225, China;

2. College of Light Industry and Food Engineering, Guangxi University, Nanning 530004, China;

3. School of public health, Guangxi Medical University; Nanning 530021, China;

4. Institute of nutrition and health, China Center for Disease Control and Prevention; Beijing 100050, China;

5. Key Laboratory of Environmental Exposomics and Entire Lifecycle Health, Guilin Medical University; Guilin 541199, China.

[+] These authors contributed equally.

* Correspondence: songjiale@glmc.edu.cn; Tel.: +86-18178360021 (J.-L.S.); liquanyang@gxu.edu.cn; Tel.: +86-13667883719 (Q.L.);

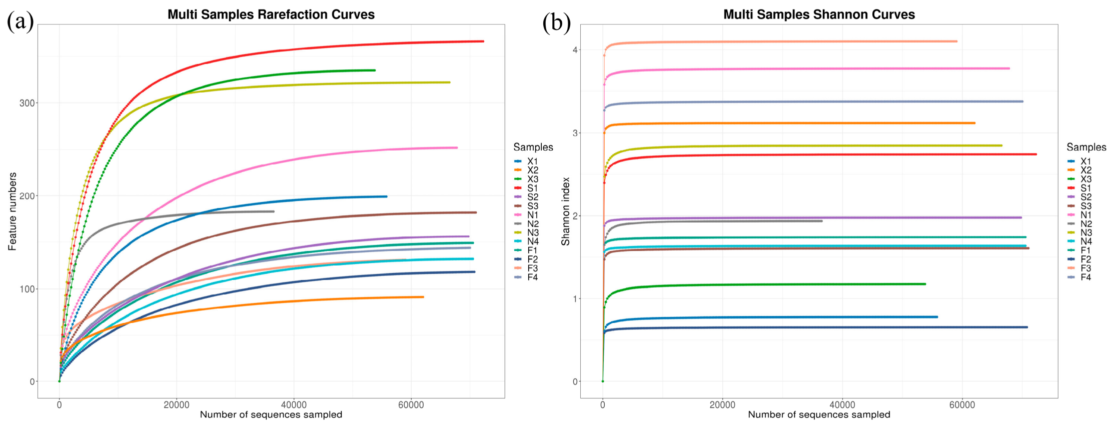

Figure S1. Dilution curve (a) and flavor curve (b) of 14 acid congee samples

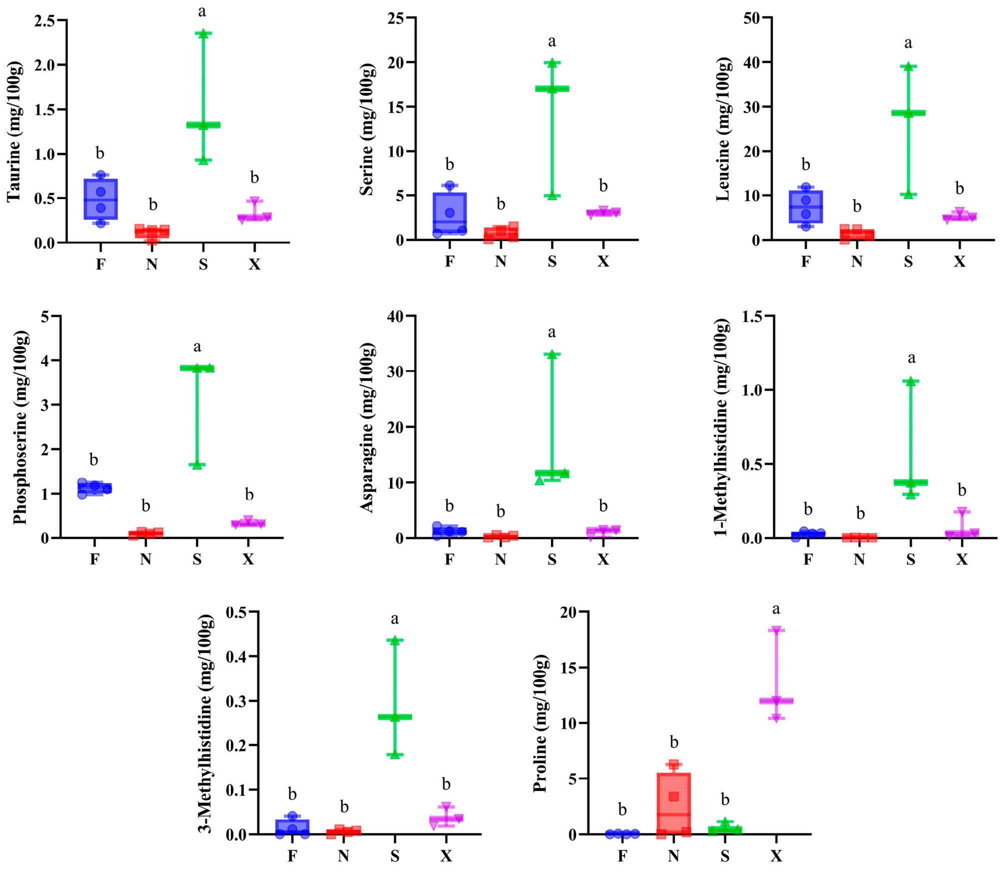

Figure S2. Amino acids with significant differences among the four groups

Table S1. 16S rRNA sequencing results of 14 acid Congee samples and the number of different classification states

No.	Reads	OTUs	Phylum	Class	Order	Family	Genus	ACE	Chao 1	Simpson	Shannon
F1	70593	149	19	35	65	92	110	149.84	149.08	0.47	1.74
F2	70886	118	19	30	57	79	94	119.11	118.07	0.17	0.65
F3	59115	131	11	16	42	60	81	134.55	131.53	0.91	4.10
F4	70200	144	22	33	64	88	106	146.62	145.08	0.86	3.37
Average ± SD	67699 ± 5729	136 ± 14	18 ± 5	29 ± 9	57 ± 11	80 ± 14	98 ± 13	137.53 ± 13.93	135.94 ± 14.08	0.60 ± 0.35	2.47 ± 1.56
N1	67789	252	21	35	75	122	150	252.87	252.06	0.88	3.77
N2	36630	183	20	35	75	108	131	183.33	183.00	0.40	1.94
N3	66633	322	22	43	95	147	194	322.39	322.10	0.60	2.85
N4	70527	132	17	33	68	95	112	132.58	132.03	0.48	1.67
Average ± SD	60395 ± 15927	222 ± 83	20 ± 2	37 ± 4	78 ± 12	118 ± 12	147 ± 35	222.79 ± 82.70	222.30 ± 82.74	0.59 ± 0.21	2.56 ± 0.95
S1	72269	366	27	50	107	175	237	366.95	366.25	0.62	2.74
S2	69795	156	17	31	58	87	105	156.30	156.00	0.53	1.98
S3	71162	182	20	36	77	106	123	182.44	182.04	0.38	1.61
Average ± SD	71075 ± 1239	235 ± 114	21 ± 5	39 ± 10	81 ± 25	123 ± 46	155 ± 72	235.23 ± 114.82	234.76 ± 114.61	0.51 ± 0.12	2.11 ± 0.58
X1	55865	199	21	35	78	112	136	200.33	199.45	0.14	0.78
X2	62103	91	11	18	31	41	56	92.84	91.33	0.80	3.11
X3	53757	335	21	39	96	146	203	335.21	335.00	0.20	1.18
Average ± SD	57242 ± 4340	208 ± 122	18 ± 6	31 ± 11	68 ± 34	100 ± 54	132 ± 74	209.46 ± 121.44	208.59 ± 122.09	0.38 ± 0.36	1.69 ± 1.25

Table S2. The α - diversity index in acid congee samples

Groups	ACE	Chao1	Shannon	Simpson
F	137.53 ± 13.93	135.94 ± 14.08	2.47 ± 1.56	0.60 ± 0.35
N	222.79 ± 82.70	222.30 ± 82.74	2.55 ± 0.97	0.59 ± 0.21
S	235.23 ± 114.82	234.76 ± 114.61	2.11 ± 0.58	0.51 ± 0.12
X	209.46 ± 121.44	208.60 ± 122.09	1.69 ± 1.25	0.38 ± 0.37

Table S3. Anova Post-hoc tests

**Component**	**F value**	***P* value**	**-LOG10(p)**	**FDR**
Pro	20.662	0.0001	3.881	0.003
PSer	18.452	0.0002	3.674	0.003
3-Met-His	16.499	0.0003	3.473	0.004
Tau	9.9347	0.002	2.618	0.019
Leu	8.0322	0.005	2.292	0.029
Ser	7.9223	0.005	2.272	0.029
Asn	7.4456	0.007	2.181	0.030
1-Met-His	6.4514	0.011	1.979	0.042
